# GBS-based identification of *Triticum timopheevii* introgressions in a wheat pre-breeding population

**DOI:** 10.3389/fpls.2026.1887191

**Published:** 2026-08-05

**Authors:** Eszter Gaál, Andrea Uhrin, Balázs Kalapos, Kateřina Holušová, Miklós Álmos Pance, Andrea Gulyás, Norbert Hidvégi, Jan Bartoš, Klaudia Kruppa, Edina Türkösi, Dimitar Douchkov, Moozhan Serpoush, László Ivanizs, András Farkas, Éva Szakács, István Molnár, Péter Mikó

**Affiliations:** 1 Hungarian Research Network (HUN-REN), Centre for Agricultural Research, Agricultural Institute, Martonvásár, Hungary; 2 Institute for Experimental Botany of the Czech Academy of Sciences, Centre of Plant Structural and Functional Genomics, Olomouc, Czechia; 3 Leibniz Institute of Plant Genetics and Crop Plant Research (IPK), Gatersleben, Germany; 4 Research and Development Institute for Wildlife and Mountain Resources, Miercurea Ciuc, Romania

**Keywords:** agronomic performance, cytogenetic analyses, genotyping-by sequencing (GBS), introgression, pre-breeding, resistance analysis, *Triticum timopheevii*, wheat

## Abstract

*Triticum timopheevii* (2n = 4x = 28, GGA^t^A^t^) is a valuable wild wheat relative for improving disease resistance and agronomic performance in bread wheat, although the detection of introgressed chromosomal segments remains challenging due to close genomic similarity. A wheat × *T. timopheevii* pre-breeding population was analyzed using Genotyping-by-sequencing (GBS) combined with a skim-seq pipeline to identify and characterize *T. timopheevii* introgressions. Read coverage analysis based on a combined *T. aestivum–T. timopheevii* reference genome enabled high-resolution detection of major chromosomal introgressions and copy-number changes. Multiple large-scale chromosomal rearrangements were identified, most frequently involving wheat chromosome 2B and *T. timopheevii* chromosome 2G, involving the replacement of a ~625 Mb wheat segment with a ~526 Mb *T. timopheevii* segment. Additional rearrangements included a 6A–6A^t^ translocation, a nullisomic 6B genotype, and widespread 1B.1R rye introgressions. These findings were confirmed by fluorescence *in situ* hybridization. Phenotyping of plant-pathogen interactions showed that some lines carrying 2G introgressions displayed reduced disease symptoms against individual leaf or yellow rust isolates. Several genotypes also showed favorable yield-component traits, including increased spikelet and grain number. Our results demonstrate that GBS-based read coverage analysis combined with cytogenetic validation is an effective approach for dissecting genome structure and identifying candidate introgressions with potential breeding relevance. The recurrent involvement of specific chromosomes highlights preferential homoeologous recombination patterns and supports the targeted utilization of *T. timopheevii* genetic diversity for wheat improvement.

## Introduction

Wild relatives of hexaploid (2n = 6x = 42, BBAADD) bread wheat (*Triticum aestivum* L.) represent an important reservoir of genetic variation for improving resistance to biotic and abiotic stresses and enhancing the sustainability of wheat production. Among them, *Triticum timopheevii* Zhuk. is a wild allotetraploid species (2n = 4x = 28, GGA^t^A^t^) belonging to the secondary gene pool of wheat (Friebe et al., 1996). It has been widely recognized as a valuable source of resistance to several diseases, including leaf and stem rust (Brown-Guedira et al., 2003; Leonova et al., 2004; McIntosh and Gyárfás, 1971), yellow rust (Badaeva et al., 2000), powdery mildew (Järve et al., 2000), smut (Belea, 1992; Järve et al., 2002), and Fusarium head blight (Malihipour et al., 2016; Steed et al., 2022), as well as tolerance to drought and salinity (Badridze et al., 2009; Colmer et al., 2006). In addition, *T. timopheevii* possesses favorable compositional quality traits such as high grain protein and mineral content, making it an attractive donor for wheat improvement (Hu et al., 2013, 2017). Numerous resistance genes originating from *T. timopheevii* have been identified and transferred into bread wheat, including genes conferring resistance to rusts (*Sr36, Sr37, Lr18, Lr50, LrTt1, LrTt2*) and powdery mildew (*Pm6, Pm27*) located mostly on G-genome chromosomes (Badaeva et al., 2000; Bai et al., 1998; Brown-Guedira et al., 2003; Leonova et al., 2010; McIntosh and Gyárfás, 1971; Yamamori, 1994). Furthermore, the cytoplasm of *T. timopheevii* underlies the T-type cytoplasmic male sterility (CMS) system, which plays a crucial role in hybrid wheat breeding by enabling controlled cross-pollination and hybrid seed production (Geyer et al., 2018; Melonek et al., 2021).

*T. timopheevii* carries the A^t^ and G genomes, which are homologous but sufficiently divergent from the A and B genomes of bread wheat to provide a rich source of novel genetic variation. This genomic similarity facilitates the introgression of beneficial traits through interspecific hybridization, and also affects recombination behaviour and the transmission of alien chromatin (Dvořák et al., 1993; Li et al., 2022; Tsunewaki and Ogihara, 1983). Commonly used cytogenetic methods, such as fluorescence and genomic *in situ* hybridization techniques (FISH and GISH) are powerful tools for detecting and identifying alien chromatin in the wheat genome; however, they are time-consuming and low-throughput techniques. Moreover, their application is limited in the case of *T. timopheevii*. Due to the high similarity between the G and B, as well as the A^t^ and A genomes, these genomes cannot be reliably distinguished using GISH (King et al., 2022). Similarly, frequently used FISH probes (Afa, pSc, and pTa71) produce highly similar hybridization patterns, making accurate chromosome discrimination difficult. Therefore, translocations and deletions - particularly small rearrangements - are difficult to confirm solely by FISH and/or GISH. Molecular markers offer an alternative approach to identify wheat- *T. timopheevii* translocations, including PCR-based markers (Ji et al., 2007), or SNP markers (Devi et al., 2019). However, these methods typically target only small genomic regions and therefore provide a limited resolution.

Advances in next-generation sequencing (NGS) technologies have enabled cost-effective, high-throughput genotyping based on genome-wide SNP markers, greatly accelerating genetic research and breeding applications. The availability of reference genomes greatly facilitates the application of high-throughput genotyping techniques. The completion of the wheat reference genome (IWGSC, 2018) enabled the detection of chromosomal introgressions by mapping short-read sequencing data to the wheat genome, without requiring a reference genome of the donor species (Keilwagen et al., 2022). The subsequent sequencing of the *T. timopheevii* genome by Grewal et al. (2024) has further improved the identification and characterization of *T. timopheevii* introgressions, as only short-read sequencing data are required and *de novo* assembly of the introgression lines is unnecessary. These genomic resources have enabled the application of reduced-representation genome sequencing techniques (Davey et al., 2011). Reduced-representation approaches such as genotyping-by-sequencing (GBS) are now widely used for diversity analysis, trait mapping, and selection in crop species, and are highly effective for detecting larger introgressions, deletions, and translocations (He et al., 2014; Scheben et al., 2017). Several studies have applied GBS, SNP arrays, and chromosome-specific markers to investigate genetic diversity and introgression from *T. timopheevii* into wheat. For example, a genome-wide association study (GWAS) revealed the widespread presence of *T. timopheevii*-derived segments across the experimental wheat panel, as well as extensive recombination between the A^t^ and G genomes and the A and B genomes of bread wheat, including loci associated with grain quality traits (Leonova et al., 2023). Yadav et al. (2023) used GBS and identified chromosomal segments associated with leaf rust resistance, while King et al. (2022) developed chromosome-specific KASP markers for analyzing wheat-*T. timopheevii* introgression lines. Cytogenetic and genomic analyses further demonstrate considerable genetic diversity within the *Timopheevii* group and its potential for wheat improvement (Peng et al., 2023). A set of SNP markers was reported by Devi et al. (2019), enabling the detection of *T. timopheevii* introgressions in a hexaploid wheat background and providing a potentially valuable tool for marker-assisted selection in wheat pre-breeding programs.

GBS analysis enabled reliable discrimination between *T. timopheevii* and *T. turgidum* L., the tetraploid (BBAA) ancestor of bread wheat revealing several morphological misclassifications and thereby emphasizing the superiority of molecular approaches for accurate species identification (Czajkowska et al., 2019). Following the approach described by Adhikari et al. (2022) introgressed genomic regions can be detected by aligning sequencing reads to a combined reference assembly of the parental genomes and evaluating normalized read coverage. This method enables inference of genome composition and copy number variation, and has been applied in several crops, including wheat, rice, chickpea, and *Thinopyrum* (Adhikari et al., 2022; Bayer et al., 2015; Kruppa et al., 2025).

Despite these advances, comprehensive characterization of introgressed segments in diverse pre-breeding populations—particularly using reduced-representation sequencing approaches combined with field-based phenotyping—remains limited.

The aim of the present study was to analyze the genomic and phenotypic characteristics of a diverse wheat pre-breeding population comprising 42 pre-breeding back-cross lines potentially carrying *T. timopheevii* chromosomal segments, together with parental wheat and *T. timopheevii* controls. The main objectives were: (1) to identify and characterize introgressed *T. timopheevii* genomic regions using GBS-based read coverage analysis and cytogenetic tools; (2) to evaluate the agronomic performance of the genotypes under field conditions, including responses to biotic and abiotic stresses; (3) to examine the genomic relationships between wheat and *T. timopheevii* chromosomes.

## Materials and methods

### Plant material

Introgression lines developed from different crossing combinations were back-crossed with elite winter wheat varieties up to 6 times (depending on the specific combinations), resulting in BC1-BC6 lines. The resulting diverse pre-breeding population comprised 42 pre-breeding lines (named according to their coordinates in the 96-well plate), potentially containing *Triticum timopheevii* genome fragments, along with 6 control genotypes (e.g., Mv9kr1 and Mv Nádor wheat and *T. timopheevii* parental lines), and was analyzed in this study. Two different *T. timopheevii* accessions were used to create this pre-breeding population: *T. timopheevii* MvGB845 (*T. timopheevii* var. *rubiginosum)* and *T. timopheevii* ssp. *armeniacum* MvGB137. For the development of interspecific hybrids, special wheat lines (Mv9kr1 and Mv Magdalénakr1) were used that carry the crossability allele in a recessive form, thereby improving seed set during interspecific crossing (Molnár-Láng et al., 1996). The detailed plant material, including pedigree information, is summarized in **Supplementary Table 1**.

### GBS library development

Genomic DNA was extracted from fresh young leaves using the BioSprint DNA kit (Qiagen GmbH, Hilden, Germany) in 96-well plates, following the manufacturer’s instructions. The double-digest restriction site-associated DNA (ddRAD) library was prepared based on the protocol by Poland et al. (2012). The main difference was the rare cutter enzyme: in this protocol SphI-HF (5’-GCATG/C-3’, New England BioLabs, Ipswich, MA, USA) was used. P1 barcoded and P2 common adapters corresponding to the restriction sites were ligated using T4 ligase (Promega, Madison, WI, USA) at 23 °C for 120 minutes, and the enzymes were subsequently inactivated at 65 °C for 20 minutes. Samples with the same P2 and 12 different P1 adapters were pooled and purified using 1.5× AMPure XP Bead-Based Reagent (Beckman Coulter, Brea, CA, USA). Size selection was performed with a dye-free BluePippin 1.5% gel cassette (BDF1510) (Sage Science, Beverly, MA, USA), targeting fragments of 350–390 bp. Illumina flow cell annealing sequences, multiplexing indices, and sequencing primer annealing regions were added to the fragments through PCR amplification using a Q5 Polymerase kit (New England BioLabs, Ipswich, MA, USA), which also increased the concentrations of the sequencing libraries. For large-scale combinatorial multiplexing, eight uniquely indexed PCR2 primers were employed, allowing each primer to add a unique index sequence to all fragments, enabling unique labelling of up to 96 individuals (12 P1 adapter barcodes × 8 PCR2 indices) in a single sequencing lane. PCR enrichment was conducted with 10 cycles in 25 μL reactions containing 1.25 μL each of NEBNext^®^ Index and Universal Primers, 0.25 μL Q5 DNA polymerase, 0.5 μL dNTPs, 0.5 μL buffer, and 16.75 μL of the template. Each sublibrary was purified using 0.9× AMPure XP Bead-Based Reagent (Beckman Coulter, Brea, CA, USA) and quantified using a Qubit fluorometer (Thermo Fisher Scientific, Waltham, Massachusetts). All sublibraries were pooled in equimolar amounts and sequenced on the NovaSeq 6000 platform (Illumina, Inc., San Diego, CA, USA) using the SP reagent kit v1.5 in a 2 × 150 bp configuration at the Institute of Experimental Botany of the Czech Academy of Sciences, Olomouc, Czech Republic.

### GBS read coverage analysis

Following the normalized read coverage approach described by Adhikari et al. (2022), for interspecific mapping a reference assembly could be constructed by concatenating the sequences of the donor and recipient species as “*in silico* interspecific hybrid”. Sequencing reads were aligned to this combined assembly, and chromosome identity and physical position could be extracted. An “*in silico* wheat × *T. timopheevii* hybrid” reference genome was constructed by combining the reference sequences of the donor and the recipient species. To identify wheat-*T. timopheevii* introgressions, we combined the Chinese Spring reference genome (IWGSC RefSeq v2.1) (Zhu et al., 2021) with the draft genome assembly of *T. timopheevii* (GCA_963921465.1) (Grewal et al., 2024). During the assembly process, unique identifiers were assigned to all chromosomes or pseudomolecules to maintain distinctiveness.

Prior to alignment, the Illumina short reads from 42 lines, along with the previously described control genotypes, were demultiplexed and adapter-trimmed with Stacks v2.68 (Rochette et al., 2019). The processed paired-end reads were then mapped to the combined reference genome using HISAT v2.2.1 (Kim et al., 2019) with the – no-spliced-alignment and – no-unal parameters. Following alignment, concordant unique reads were retrieved by filtering the sequence alignment map (SAM) outputs for the YT:Z:CP and NH:i:1 tags. For data analysis and visualization, total read counts were computed per 1 Mb genomic bins across all chromosomes. while reads mapped to unknown chromosomes were removed. These read counts were subsequently normalized across the 1 Mb window. The normalized read counts were computed as:


$$\frac{normalized\;reads}{Mb}=\frac{sum\;of\;reads\;in\;Mb\;bin}{total\;number\;of\;reads\;per\;sample}\times Normalization\;Factor$$

based on Adhikari et al. (2022). Graphical representation of these normalized values enabled the visualization of introgressed segments and copy number variation across both wheat and alien chromosomes. Relative read count values close to 0 indicate the absence of a genomic region (deletion); values around 0.5 suggest monosomic presence; values near 1 correspond to disomic presence; and higher values reflect increased copy number. The custom scripts used for this data analysis are available at https://github.com/sandeshsth/SkimSeq_Method. Data analysis was conducted using a high-throughput skim-seq pipeline, following the protocol outlined by Adhikari et al. (2022).

### Molecular cytogenetic analysis

Seedlings were germinated and grown in a hydroponic system. To synchronize cell division in root tip meristems, hydroxyurea was added to the Hoagland solution, followed by amiprophos-methyl treatment to accumulate cells at metaphase (Doležel et al., 1992; Vrána et al., 2016). Chromosome spreads were made using the air-dry drop method as described by Kato et al. (2004). The probe labelling and fluorescence *in situ* hybridization (FISH) on selected genotypes, followed the protocol outlined by Mikó et al. (2015). In summary, the pSc119.2 and Afa-family sequences were amplified and labelled with biotin-16-dUTP (Roche Diagnostics, Mannheim, Germany) and digoxigenin-11-dUTP (Roche Diagnostics, Mannheim, Germany), respectively, using PCR (Contento et al., 2005; Nagaki et al., 1995). Digoxigenin and biotin labelled oligo-pTa71 probe was synthesized by IDT (Coralville, IA, USA) and combined in a 6:5 ratio to detect a yellow signal (Tang et al., 2014). Digoxigenin- and biotin-labelled probes were detected using anti-digoxigenin-rhodamine Fab fragments (Roche Diagnostics, Mannheim, Germany) and Alexa Fluor-488 streptavidin conjugate (Invitrogen, Life Technologies, Carlsbad, USA), respectively. Chromosomes were counterstained with 2 μg/ml DAPI (4’,6-diamidino-2-phenylindole, Amersham Biosciences, Piscataway, NJ) and fluorescence signals were visualized using a Zeiss Axio Imager Z2 upright epifluorescence microscope (Carl Zeiss, Jena, Germany). Images were captured with a MetaSystems CoolCube 4 USB Laboratory Camera and analyzed using Metafer 4 (automatic metaphase image capture) and ISIS (image processing) software (MetaSystems, Altlussheim, Germany).

### Phenotyping of plant-pathogen interactions

#### Macrophenotyping

The macrophenotypic data were collected using the Macrobot Platform at IPK Gatersleben, which enables high-throughput, semi-automated detached leaf-assay (Lück et al., 2020b). A comprehensive description of the experimental conditions and inoculation procedures is described by Hinterberger et al. (2023). Wheat stripe rust (*Puccinia striiformis* f. sp. *tritici*, isolate PstS10) and wheat leaf rust (*Puccinia triticina*, isolate PD23) responses were assessed using a detached-leaf assay. Plants were grown under greenhouse conditions, and the second leaves were collected from two-week-old seedlings. Leaf segments of 2 cm length were cut and placed on 1% water agar supplemented with 35 mg L⁻¹ benzimidazole as a senescence inhibitor and 1 mg L⁻¹ AgNO_3_ to suppress bacterial growth.

Leaf segments were inoculated with dry uredospores in an inoculation tower by blowing spores onto the leaf surface at a density of approximately 3 to 5 spores per mm². Spore density was monitored by counting spores deposited on reference slides placed between the samples. After inoculation, plates were transferred to controlled-environment incubators shortly before the onset of the night cycle. Infected leaves were incubated for 14 days under a 16 h light/8 h dark photoperiod, with day/night temperatures of 16 °C/12 °C and 80% relative humidity. Disease symptoms were subsequently quantified using the automated macro-phenotyping platform Macrobot, as described by (Lück et al., 2020b).

The experiment was conducted in three independent replicates, with eight plants per genotype in each replicate.

After incubation, high-resolution images (3,296 × 2,472 pixels) were captured using a monochrome camera under sequential illumination with different wavelengths corresponding to red, green, blue, and UV light. The images were stored in TIFF format. Details of the hardware setup are described in Lück et al. (2020b). The percentage of infected leaf area was then quantified from the image data using an open-source Python-based algorithm (Lück et al., 2020a).

#### Data curation of phenotypic data

Disease severity was analyzed as the percentage of infected leaf area. Best linear unbiased estimates (BLUEs) for each genotype were calculated using a linear mixed model in R with the lme4, lmerTest, and emmeans packages. Genotype was treated as a fixed effect, while experiment and inoculation group nested within experiment were included as random effects. The model used was:

infected leaf area ~ genotype + (1 | experiment) + (1 | experiment:inoculation group).

BLUEs were extracted as estimated marginal means for each genotype.

### Field phenotyping experiments

The field phenotyping trial was conducted in a high-input breeding nursery of HUN-REN Centre for Agricultural Research, Agricultural Institute at Martonvásár, Hungary, (geographic coordinates: 47°19′51″N, 18°47′01″E) in the 2022–2023 winter wheat growing season. Cultivars were grown in chernozem soil that is suitable for wheat cultivation. Each genotype was sown in 2 m² plots (6 rows of 2 m length; 50 grains per row) with a row spacing of 0.15 m. Detailed growth parameters can be found in **Supplementary Table 2**. Experimental plots were sown using a HEGE-80 line driller machine, and harvest was conducted manually. Fertilizers, herbicides, and insecticides were applied in accordance with conventional Hungarian wheat-growing practice, but fungicides were not used. To minimize the confounding effects of soil and microclimatic variables, genotypes were sown in close proximity. Ten inner plants per genotype were randomly selected from each plot for the characterization of the following morphological traits: plant height (cm), number of seeds per spikelet, length of the main spike (cm), number of seeds per main spike, number of spikelets per main spike and number of seeds per plant. Plant height and tillering (number of spikes per plant) were recorded in the field right before harvest, while the other yield-related characteristics were assessed after harvest. Exploratory comparisons between pre-breeding lines and controls were performed using two-sample t-tests based on plant-level measurements. The analysis was performed on data from 10 sampled plants per genotype.

## Results

### GBS read coverage analysis

We applied GBS technology to identify potential chromosomal deletions and rearrangements, and to determine the homoeologous relationship of the introgressed *T. timopheevii* chromosomes to wheat. The quality-filtered and demultiplexed read data were aligned to the combined *T. aestivum*-*T. timopheevii* reference genome, and reads with a unique map position were used for normalized read coverage analysis.

The total raw read count for the samples analyzed in this manuscript: 324 179–896 raw reads (162 089–948 read pairs). Based on the sequencing configuration (2 × 150 bp), this corresponds to approximately 48.63 Gb of raw sequencing data for the samples included in this study. The GBS read coverage analysis provided clear evidence of the genomic constitution in the parental lines (**Table 1**). The wheat reference parent (Mv9kr1), representing the hexaploid wheat (BBAADD) genome, showed high and uniform read coverage across the A, B, and D genome chromosomes (ranging from 155.8 to 237.9 reads/Mb), with minimal read depth on the A^t^ and G chromosomes (4.8 to 16.2 reads/Mb), confirming its pure hexaploid wheat (BBAADD) background. In contrast, *T. timopheevii* displayed strong coverage on the A^t^ and G chromosomes (86.0–101.5 reads/Mb) and negligible coverage on wheat chromosomes (<1 read/Mb), consistent with its GGA^t^A^t^ genomic constitution.

**Table 1:** Genome-specific distribution of sequencing reads from *Triticum aestivum* ‘Mv9kr1’ and *T. timopheevii* mapped to a *T. aestivum–T. timopheevii in silico* hybrid reference.

*In silico* chromosome	Chromosome size in Mb	Chromosome coverage (%)	Mv9kr1		*T. timopheevii*	
			Number of reads	Average read/Mb	Number of reads	Average read/Mb
1A	598	100,0	93142	155,8	412	0,7
2A	787	99,9	124872	158,7	1098	1,4
3A	754	99,9	127892	169,6	480	0,6
4A	754	100,0	136184	180,6	428	0,6
5A	713	100,0	116004	162,7	622	0,9
6A	622	100,0	99100	159,3	310	0,5
7A	744	100,0	123668	166,2	772	1,0
1B	700	99,9	123194	176,0	208	0,3
2B	812	99,8	143860	177,2	290	0,4
3B	851	99,8	152994	179,8	400	0,5
4B	673	100,0	133508	198,4	156	0,2
5B	714	100,0	136762	191,5	246	0,3
6B	731	99,7	141660	193,8	416	0,6
7B	763	100,0	148804	195,0	338	0,4
1D	498	100,0	107046	215,0	86	0,2
2D	656	99,4	136520	208,1	426	0,6
3D	619	100,0	140214	226,5	70	0,1
4D	518	100,0	152256	293,9	100	0,2
5D	569	100,0	128072	225,1	114	0,2
6D	495	100,0	111478	225,2	86	0,2
7D	642	100,0	152706	237,9	108	0,2
1A_t_	614	85,5	8706	14,2	53330	86,9
2A_t_	767	90,7	11910	15,5	67502	88,0
3A_t_	669	80,3	6402	9,6	56666	84,7
4A_t_	770	84,3	10958	14,2	69850	90,7
5A_t_	693	84,8	9822	14,2	59592	86,0
6A_t_	585	82,7	6646	11,4	55508	94,9
7A_t_	745	86,0	12100	16,2	66122	88,8
1G	493	72,8	3052	6,2	46258	93,8
2G	669	76,8	7588	11,3	64606	96,6
3G	669	76,1	9240	13,8	65486	97,9
4G	642	67,4	3106	4,8	59610	92,9
5G	640	72,7	4546	7,1	62210	97,2
6G	588	73,1	4890	8,3	56942	96,8
7G	692	73,8	4118	6,0	70220	101,5

Based on the validated genomic profiles of the parental lines, the analysis was extended to the pre-breeding population to assess genome composition and introgression patterns.

**Figure 1:**
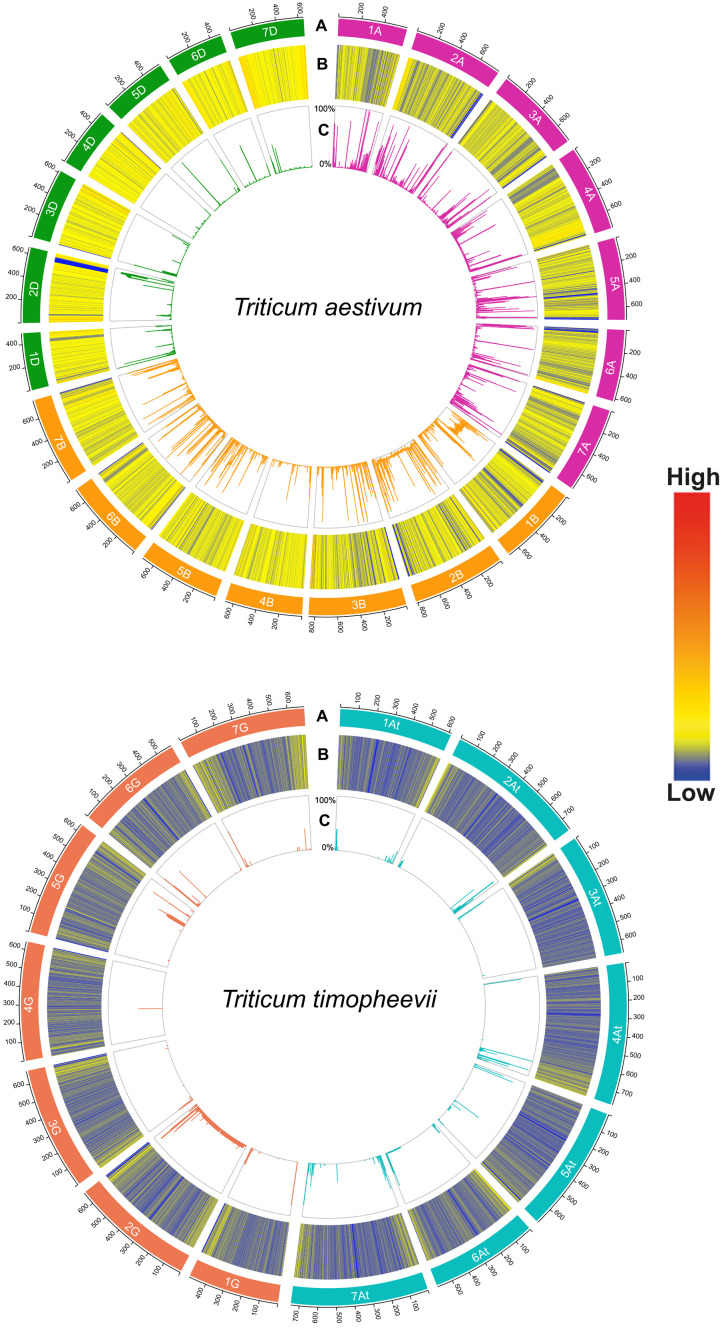
Circos plot depicting the distribution of GBS reads across the population mapped onto the wheat (*Triticum aestivum* (top) and *T. timopheevii* (bottom) genomes. The outermost **(A)** tracks represent the chromosomes of wheat **(A, B, D** subgenomes) and *T. timopheevii* (A^t^, G subgenomes), respectively. Inner heatmaps **(B)** reflect the normalized read density (blue to red, low to high), while the histograms **(C)** show the distribution (0-100%) of GBS read depth along each chromosome. GBS reads derived from Mv9kr1 wheat were mapped to a combined *T. aestivum*–*T. timopheevii* reference genome.

The wheat D genome was relatively stable in the pre-breeding population, with a low frequency of missing segments, whereas the A and B genomes showed more variable patterns (**Figure 1**). The 1BS terminal region was missing in almost half of the lines (**Figure 1**), suggesting the presence of 1B.1R translocation, which was further supported by alignment to the rye reference genome (unpublished data). This observation is consistent with the pedigree of the pre-breeding populations, as different elite wheat cultivars were used as recurrent parent in the independent pre-breeding programs. Among these, Mv Nádor and Mv Magdalénakr1 carry the 1BL.1RS rye-wheat translocation, explaining the occurrence of this segment in a substantial proportion of the analyzed lines (**Supplementary Table 1**).

The chromosomal distribution of GBS read depth showed that some *T. timopheevii* regions were more frequent in the pre-breeding population, including terminal regions of 2A^t^, 4A^t^, 5A^t^, 5G, 6G, and chromosome 2G, whereas others (3A^t^, 3G, and 4G) were largely absent (**Figure 1**).

**Figure 2:**
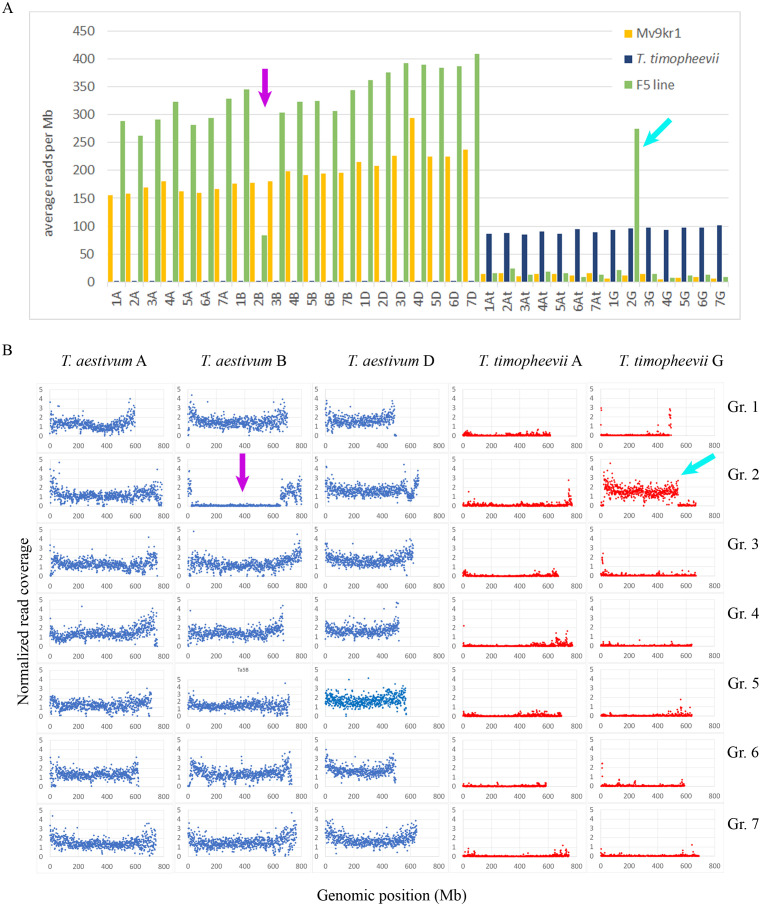
Results of the GBS read coverage analysis along the *in silico T. aestivum – T. timopheevii* hybrid chromosomes. **(A)** Average number of reads per 1 Mb of the Mv9kr1 and *T. timopheevii*, and the pre-breeding line F5. **(B)** Normalized GBS read coverage of the line F5 genotype along the A, B, D A^t^, and G chromosomes of the *in silico* hybrid. Each panel corresponds to a different wheat chromosome (indicated on the left). The x-axis represents the genomic position within the chromosome in Mb, while the y-axis shows the normalized read coverage. Purple arrows indicate the lack of the 2B segment, while turquoise arrows indicate the introgression of the 2G segment.

When a selected pre-breeding line (line F5, according to **Supplementary Table 1**) was mapped to the combined reference genome, a very high coverage across all wheat chromosomes (up to 409.1 reads/Mb on 4D) was found, indicating the retention of the full hexaploid wheat genome, except for the 2B chromosomes, which showed a lower coverage (83.8 reads/Mb) (**Figure 2A**). Additionally, low coverage was observed on the A^t^ and G chromosomes—except for the 2G (274.5 reads/Mb). This pattern indicates a 2G–2B chromosome translocation. Clear breakpoints were identified on both chromosomes 2B and 2G (**Figure 2B**), indicating a homoeologous translocation. The coverage pattern revealed that the central region of wheat chromosome 2B (approximately 25–650 Mb) is largely absent, while a corresponding segment from chromosome 2G of *T. timopheevii* (approximately 19–545 Mb) is present, indicating a large-scale, non-equivalent homoeologous translocation involving the replacement of a ~625 Mb wheat chromosomal segment with a ~526 Mb segment from *T. timopheevii*.

**Figure 3:**
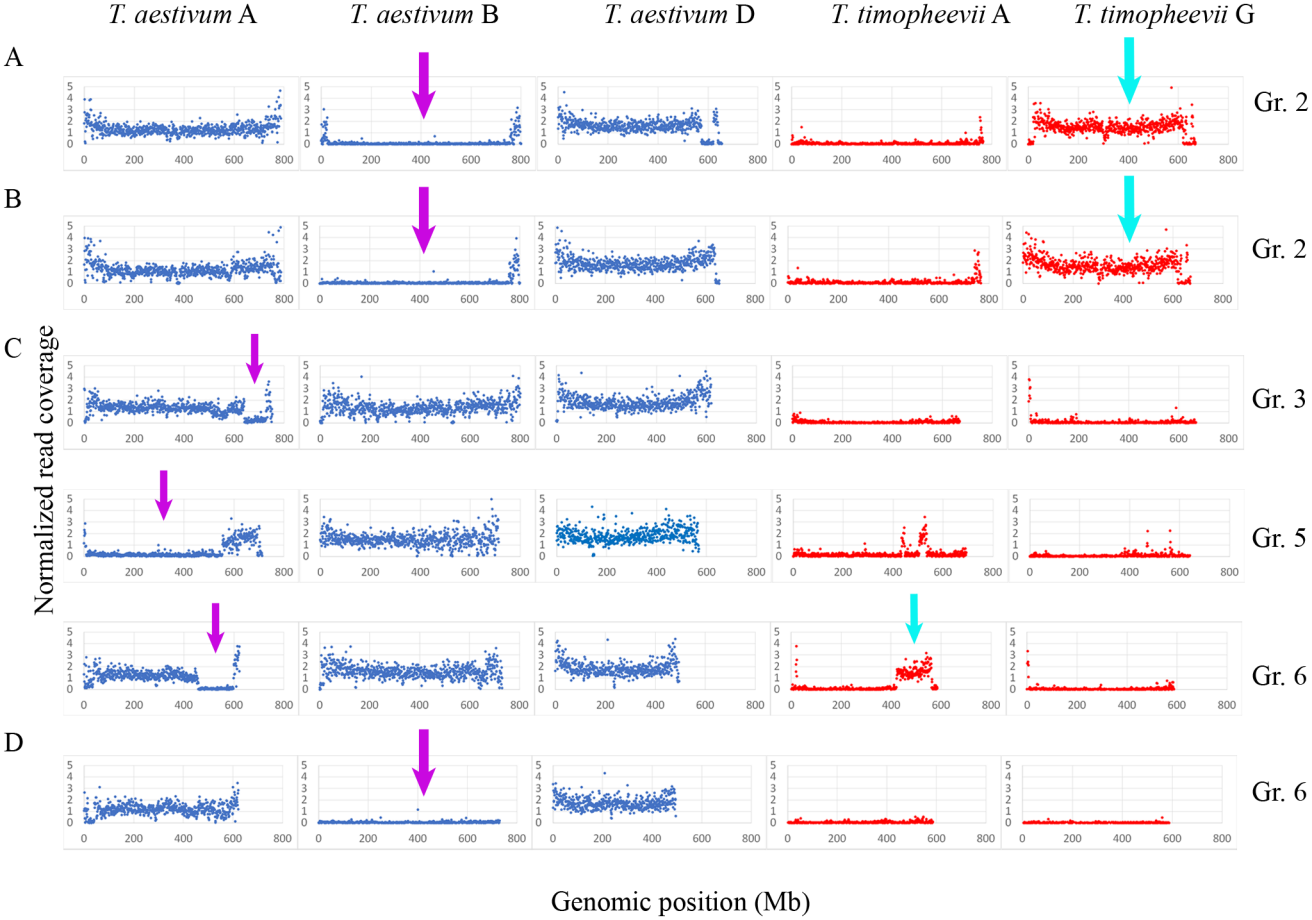
Normalized GBS read coverage of the pre-breeding lines F2 **(A)**, F9 **(B)**, F8 **(C)** and E12 **(D)** along the A, B, D, A^t^ and G chromosomes of the *in silico* hybrid. Each panel corresponds to a different wheat chromosome (indicated on the right). The x-axis represents the genomic position within the chromosome in Mb, while the y-axis shows the normalized read coverage in the case of the indicated chromosome. Purple arrows indicate the lack of the wheat segments, while turquoise arrows indicate the introgression of the *T. timopheevii* segments.

Based on the GBS read coverage analysis, four pre-breeding lines carrying major translocations from *T. timopheevii* were identified: three (F5, F2, F9) involved wheat chromosome 2B and *T. timopheevii* chromosome 2G (**Figures 3A, B**). One line (E12) carrying a translocation involving wheat 6A and *T. timopheevii* 6A^t^, also exhibited deletions on 5A (9–554 Mb) and 3AL (641–727 Mb) (**Figure 3C**).

Another line, F8, was found to be an aneuploid genotype carrying 40 chromosomes, as it completely lacked the wheat chromosome 6B (**Figure 3D**).

**Figure 4:**
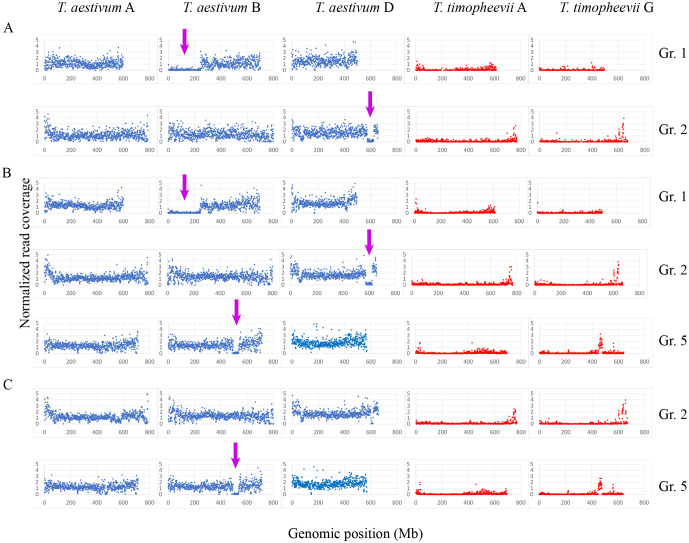
Normalized GBS read coverage of the pre-breeding lines A7 **(A)**, D7 **(B)** and C3 **(C)** along the A, B, D, A^t^ and G chromosomes of the *in silico* hybrid. Each panel corresponds to a different wheat chromosome (indicated on the left). The x-axis represents the genomic position within the chromosome in Mb, while the y-axis shows the normalized read coverage in the case of the indicated chromosome. Purple arrows indicate the lack of the wheat segments.

A total of 18 pre-breeding lines exhibited a 1B.1R rye translocation (0–245 Mb) (**Figures 4A, B**). A deletion on the long arm of chromosome 2D (575–622 Mb) was detectable in almost every line (**Figures 4A, B**). A small deletion on the long arm of chromosome 5B (495–536 Mb) was identified in 17 lines. (**Figures 4B, C**).

The normalized GBS read coverage diagrams for all 42 pre-breeding lines and parental and control lines can be found in **Supplementary Figures 1**–**6**.

### Molecular cytogenetic analysis

**Figure 5:**
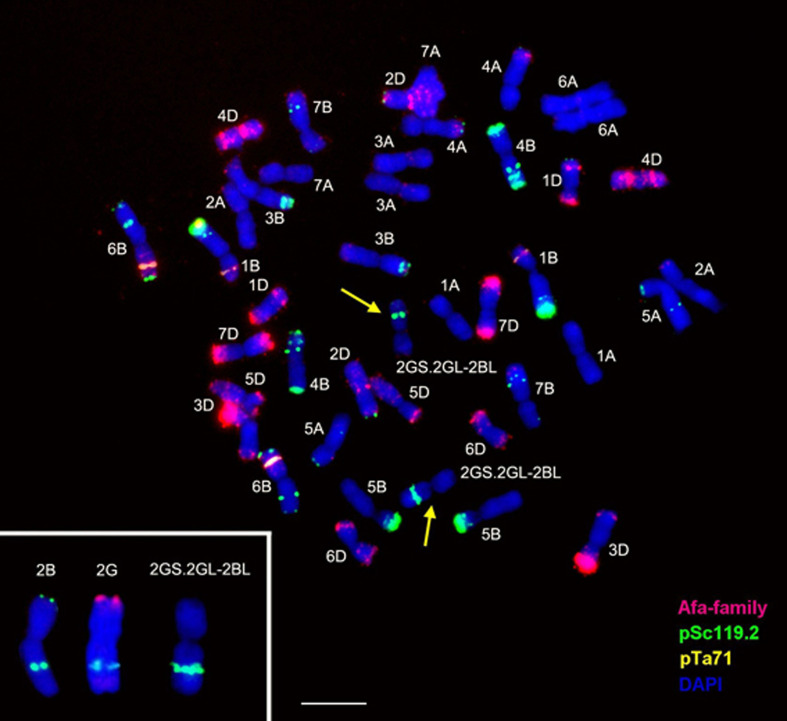
Fluorescence *in situ* hybridization (FISH) patterns of the pre-breeding line F9 belonging to the wheat – *T. timopheevii* breeding population. Repetitive DNA FISH patterns (pSc119.2, Afa-family, pTa71) showing the identification of the chromosomes, yellow arrows indicate the introgressed 2GS.2GL-2BL chromosome. At the bottom left corner a wheat 2B, a *T. timopheevii* 2G and the translocational chromosome are presented. Scale bar = 10 µm.

We performed detailed FISH analyses to investigate the chromosome constitution of the selected lines and to confirm that the translocations detected by GBS analyses were not limited to unique chromosome segments. Using repetitive DNA probes—Afa-family (red), pSc119.2 (green), and oligo-pTa71 (yellow), - in the case of three lines (F2, F5 and F9) - we were able to identify 20 wheat chromosome pairs based on their characteristic FISH signal patterns, but instead of a typical pair of 2B chromosomes, we observed a structurally altered chromosome pair with a similar but distinct hybridization pattern (**Figure 5**). According to the *T. timopheevii* karyotype Mikó et al. (2015), this chromosome pair corresponds to a translocation chromosome composed mainly of 2G and 2B segments (2GS.2GL-2BL).

In particular, in common wheat, the telomeric region of the short arm of chromosome 2B (2BS) is characterized by a pSc119.2 signal, whereas in *T. timopheevii* the corresponding region on chromosome 2G carries Afa-family repeats. However, neither signal was detected on the 2GS.2GL-2BL translocation chromosome. Based on GBS read coverage, chromosome 2G short arm is present along its full length in this line, and therefore, the presence of the Afa-family signal would be expected. The absence of both signals suggests that the terminal repeat regions characteristic of both parental chromosomes are not preserved in the translocated chromosome (**Figure 5**).

Leaf and yellow rust resistance in the *T. timopheevii* pre-breeding population.

In this work, 11 wheat- *T. timopheevii* pre-breeding lines and 7 parental/control lines were analyzed for resistance to leaf (LR) and yellow (YR) rust using the Macrobot BluVision system (for the detailed plant material see **Supplementary Table 1**). Detached leaves were infected with leaf rust (*Puccinia triticina*) isolate PD23, and yellow rust (*P. striiformis*) isolate PstS10 in a separate layout.

**Figure 6:**
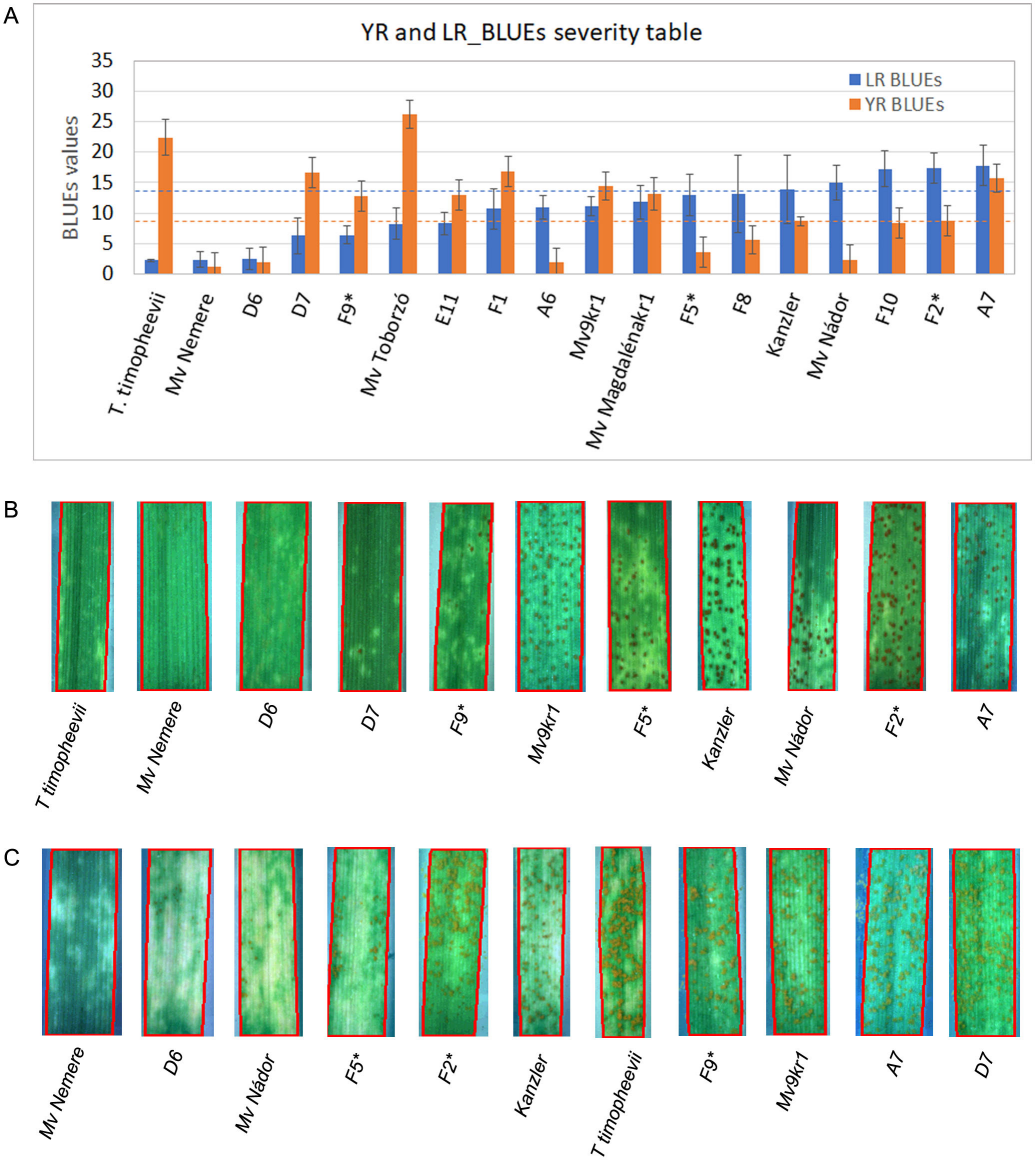
Results of the leaf rust and yellow rust resistance analyses. Tested genotypes are shown on the x-axis, and the BLUEs values on the y-axis for leaf rust (blue) and yellow rust (orange) **(A)**. The wheat cultivar Kanzler was used as the susceptible control. Dashed lines indicate the BLUEs values of Kanzler for each disease; genotypes with lower BLUE values than Kanzler showed reduced disease symptoms, whereas those with similar or higher values were classified as susceptible under these assay conditions. The translocational lines are indicated with an asterisk. Detached leaves 10 days after inoculation with leaf rust (*Puccinia triticina*) isolate PD23 are shown in **(B)**, and detached leaves 15 days after inoculation with yellow rust (*P. striiformis*) isolate PstS10 are shown in **(C)**.

Kanzler wheat was used as the susceptible control in both rust resistance analyses. The *T. timopheevii* parental line showed strongly reduced symptoms against the leaf rust isolate PD23 (**Figures 6A, B**). Among the translocation lines, F9 also showed reduced symptoms to this isolate, whereas F5 showed a response similar to the susceptible control Kanzler (**Figures 6A, B**). The pre-breeding line F2 was classified as susceptible to PD23 under the tested assay conditions (**Figures 6A, B**).

Regarding yellow rust, the *T. timopheevii* parental line was susceptible to the monosporic isolate PstS10 used in this study, whereas the elite wheat parental lines Mv Nemere and Mv Nádor showed resistance (**Figures 6A, C**). By contrast, among the translocation lines, F5 showed reduced symptoms to this isolate, while F2 and F9 were classified as susceptible under the tested assay conditions, similar to Kanzler (**Figures 6A, C**).

### Field phenotyping experiments

**Figure 7:**
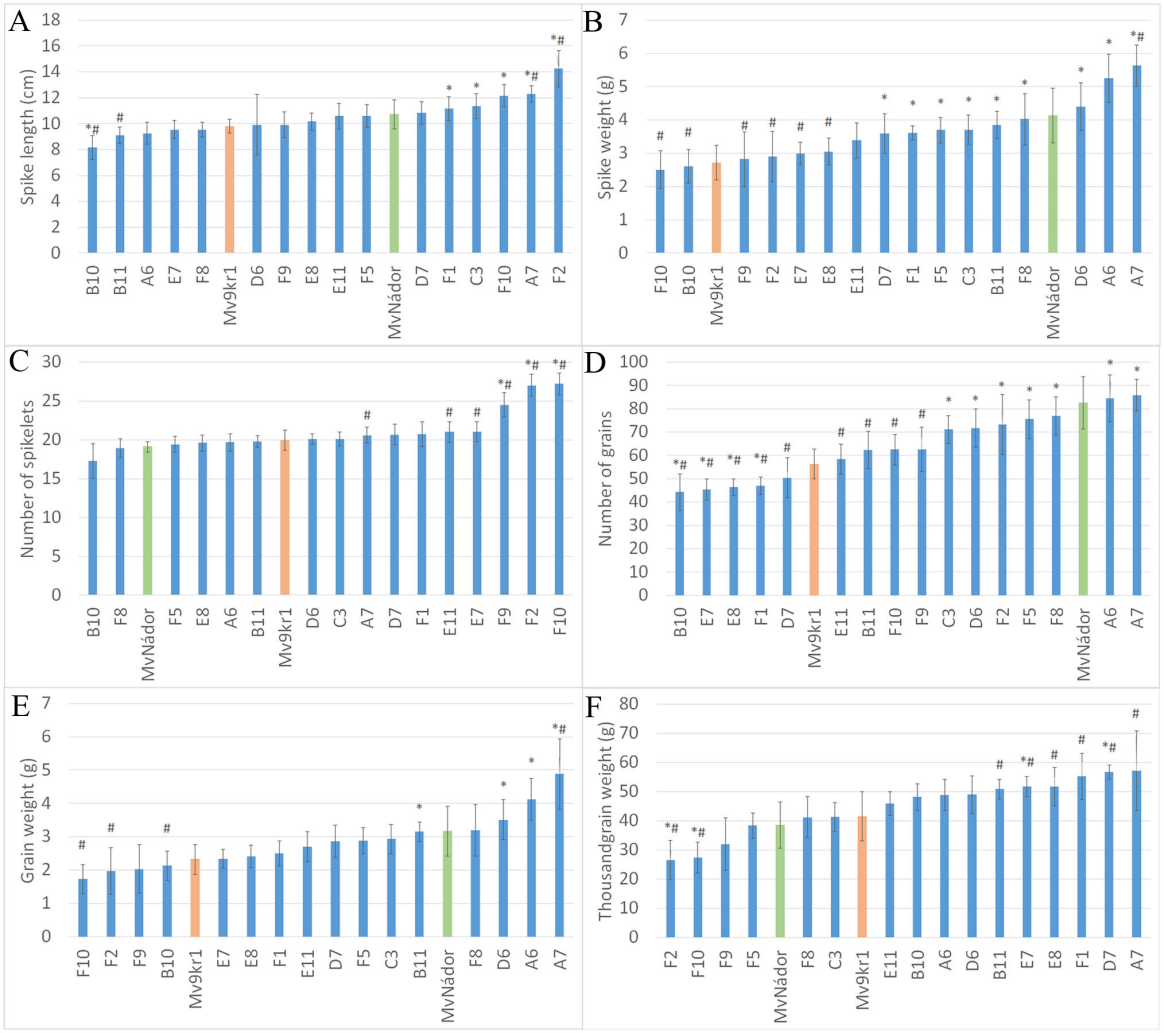
Morphological traits of the selected pre-breeding lines and their controls grown under field conditions (all parameters refer to the main spike): **(A)** spike length (cm); **(B)** spike weight (g); **(C)** number of spikelets; **(D)** number of grains; **(E)** grain weight (g); and **(F)** thousand-grain weight (g). Values are the means ± standard deviations of ten measurements. *Significantly different from the value of parental wheat Mv9kr1 at p=0.001. #Significantly different from the value of elite wheat variety Mv Nádor at p=0.001.

**Figure 8:**
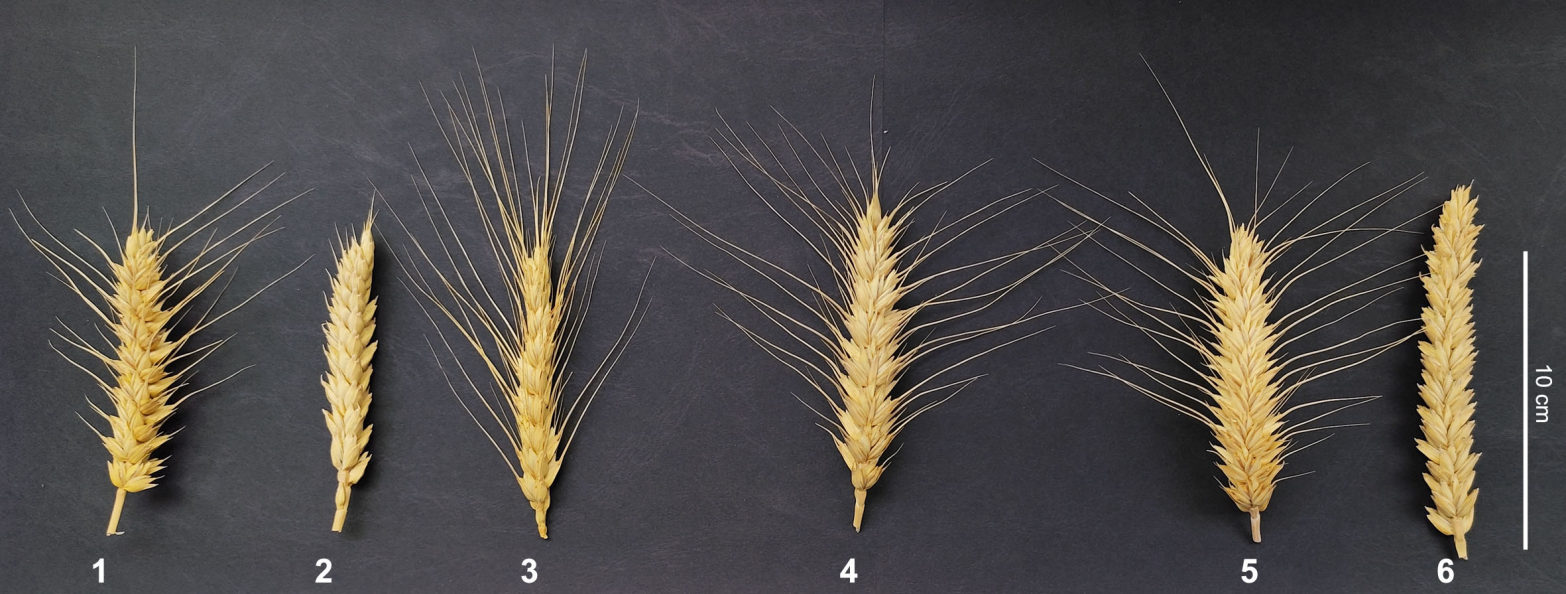
Spike morphology of selected translocation and control lines. 1) line F8, 2) Mv9kr1, 3) line F9, 4) line F5, 5) Mv Nádor, 6) line F2.

Considerable variation was observed among the genotypes in all measured traits related to main spike morphology and yield components in the single-season evaluation (**Figures 7**, **8**; **Supplementary Table 3**).

Overall, the three *T. timopheevii* translocation lines (F2, F5, F9) showed alterations in spike morphology, but these were not uniformly reflected in enhanced yield performance. Line F2 had the longest main spike (14.3 cm) and the highest number of spikelets per spike (27.0), with values significantly higher than those of both wheat parental controls, Mv9kr1 and Mv Nádor, in the exploratory plant-level comparison (**Figures 7A, C**). Line F2 had the longest main spike (14.3 cm, **Figure 8**) and the highest number of spikelets per spike (27.0), exceeding both wheat parental controls, Mv9kr1 and Mv Nádor (**Figures 7A, C**). The number of grains was also higher than that of Mvk9kr1 (**Figure 7D**). Nevertheless, reduced spike weight, grain weight, and thousand-grain weight (TGW) suggest a negative association between increased spike size and grain weight. Line F5 showed a moderate increase in main spike length (10.6 cm) and higher main spike weight (3.7 g), grain number (75.5), and grain weight per spike (2.9 g) compared to the control Mv9kr1 (**Figures 7A, B, D, E**). However, the TGW did not show an increase (38.3 g) (**Figure 7F**). Line F9 was characterized by a significantly higher number of spikelets per main spike (24.5), while other traits did not differ significantly from the control Mv9kr1 (**Figure 7C**). Grain number (62.5) and TGW (32.0 g) were relatively low, indicating limited yield advantage despite the increased spikelet number (**Figures 7D, F**).

In the case of the 6B nullisomic line (line F8), the main spike length (9.5 cm) did not differ significantly from the control; however, the main spike weight (4.0 g), grain weight (3.2 g), and grain number per main spike (76.8) were all significantly higher. The TGW (41.2 g) was comparable to that of the control.

Several genotypes, including lines A7, F10 and F1, exhibited significantly longer spikes compared to the control genotype Mv9kr1, indicating enhanced spike elongation capacity.

The line A7 exhibited strong agronomic performance, with enhanced morphological traits compared with the control line Mv9kr1. In multiple instances, its performance also exceeded that of the elite cultivar Mv Nádor (**Figures 7A–C, E, F**). These favorable traits may be associated with the presence of the 1B.1R translocation in the A7 line. Notably, *T. timopheevii*–derived translocation was not detected in this line. Lines containing 1B.1R translocation (D6, D7, B11, A6, A7) showed enhanced yield-related traits (**Figures 7D–F**), while the line F9 containing 1B.1R and 2GS.2GL-2BL translocations showed increased spikelets number, but decreased grain weight per main spike and TGW. (**Figures 7C, E, F**).

Weight of the main spike varied between 2.5 g (F10) and 5.6 g (A7). Lines A7 and A6 displayed the highest main spike weights, which were the result of the increased grain weight of their main spikes (4.9 g and 4.1 g, respectively).

The number of spikelets per main spike ranged from 17.3 (2B10) to 27.2 (2F10). Lines F2 and F10 produced the highest spikelet numbers; however, this increase was not consistently reflected in the number of grains per main spike. The highest grain number was recorded for line A7 (85.8), followed closely by A6 (84.5), indicating superior fertility and grain set efficiency in these genotypes. In comparison, the control genotype (Mv9kr1) produced 56.3 ± 6.3 grains per spike.

TGW exhibited substantial genotypic variation, ranging from 26.5 g (F2) to 57.2 g (A7). High TGW values were observed in lines A7, D7, F1, E8, and E7, indicating enhanced grain-filling capacity. Notably, some lines with high grain numbers (e.g. A7) also maintained high TGW, indicating a favorable balance between grain size and assimilate supply. In contrast, lines with high spikelet numbers but lower TGW (e.g., F2 and F10) suggest a possible trade-off between grain number and grain weight.

## Discussion

Genotyping-by-sequencing (GBS)–based read coverage analysis proved to be a powerful and high-resolution approach for dissecting the genomic constitution of wheat–*T. timopheevii* introgression lines. The clear differentiation between the parental genomes confirmed the genomic integrity of both the wheat and *T. timopheevii* parents, and revealed complex chromosomal rearrangements in the progeny.

In the selected progeny lines, GBS read coverage analysis revealed extensive chromosomal restructuring, particularly involving homoeologous group 2 chromosomes. Among the 42 analyzed pre-breeding lines, four carried major translocations involving *T. timopheevii*: three involved wheat chromosome 2B and *T. timopheevii* chromosome 2G, while one involved wheat chromosome 6A and *T. timopheevii* chromosome 6A^t^. Our result is in line with those of Devi et al. (2019), who found that the chromosome 2G was transmitted at a much higher frequency than other chromosomes from *T. timopheevii.*

The frequent occurrence of 2B–2G translocations, and the 6A–6A^t^ translocation suggest a strong homoeologous affinity between these chromosomes, consistent with previous observations of high sequence similarity between the A^t^ and A, and G and B genomes (Dvořák et al., 1993; King et al., 2022; Tsunewaki et al., 1996). 1A^t^, 2A^t^, 5A^t^, 7A^t^, 2G, 3G, 5G, and 6G do not structurally differ from the homoeologous A and B chromosomes of wheat (Badaeva et al., 2010; Maestra and Naranjo, 1999). This close relationship likely facilitates homoeologous recombination, leading to recurrent and preferential exchanges between these chromosomes. FISH analysis confirmed the presence of structurally altered chromosomes and supported the GBS-based inference of a 2GS.2GL-2BL translocation, demonstrating the complementarity of molecular and cytogenetic approaches for detecting complex chromosomal rearrangements.

Out of 42 lines, one genotype lacked the wheat 6B chromosome entirely. The line carrying the 6A–6A^t^ translocation also exhibited deletions on chromosome arms 5ASL (9–554 Mb) and 3AL (641–727 Mb), indicating structural instability in this genotype. The extensive deletions likely compromised the plant’s viability, and it could not be harvested.

A total of 18 lines exhibited a 1B.1R rye translocation (0–245 Mb), indicating that rye-derived chromatin is relatively common among the analyzed lines due to the multiple back-crossing with 1B.1R elite wheat varieties, Mv Nádor and Mv Nemere. Globally, more than 1000 wheat lines and cultivars carrying the 1BL.1RS translocation have been developed (Jiang et al., 2017; Schlegel, 2022), and are grown on over five million hectares due to their contribution to agronomic performance and disease resistance (Kumar et al., 2003). This suggests the potential utilization of some of these wheat – *T. timopheevii* pre-breeding lines not only in wheat breeding programs but also in further pre-breeding, crossing, and marker-trait validation. Based on our spike morphological results, the 1B.1R translocation may have a positive effect on the yield components. Lines containing 1B.1R translocation (D6, D7, B11, A6, A7) showed enhanced yield-related traits (number of grains, grain weight, and TGW), while the line F9 carrying 1B.1R and 2GS.2GL-2BL translocations showed increased spikelet number, but decreased grain weight and TGW. Although several introgression lines exhibited favorable spike morphology and yield-related traits, these observations were based on a single-season evaluation and therefore require multi-environment validation to assess their stability and breeding value.

The GBS analysis revealed detailed information about the parental lines. A deletion on the long arm of chromosome 2D (575–622 Mb) was detectable in the wheat parental line Mv9Kr1 and in almost every progeny line. Chen et al. (2023) identified this 596.0–614.8 Mb interval region on chromosome 2D of Chinese Spring as an exotic introgression from the N genome of *Aegilops uniaristata.* Although we identified this deletion in that region, the presence of the N-genomic fragment was not detectable by read coverage analysis (Kruppa et al., 2025). This region also corresponds to a well-known historical introgression, that has been identified in several wheat cultivars, including Vilmorin-27, for which (Keilwagen et al., 2022) suggested *Ae. markgrafii* or *Ae. umbellullata* as putative donor; however our analyses did not support *Ae. umbellulata* as the donor species (unpublished data).

A small deletion on the long arm of chromosome 5B (495–536 Mb) was identified in 17 progeny lines. This region overlaps the *Pairing homoeologous 1* (*Ph1*) locus, which regulates chromosome pairing in wheat (Dunford et al., 1995; Sears, 1976). A deletion mutant of this locus are known to promote homoeologous recombination in wheat x alien hybrids, and is used widely in wheat breeding (Copete-Parada et al., 2021; Li et al., 2020; Rey et al., 2015). Pre-breeding programs in Martonvásár frequently use the Mv9kr1*ph1* line (Türkösi et al., 2022) to induce homoeologous recombination between wheat and alien species. The successful development of the translocational lines could be due to this mutation.

Chromosome 2G from *T. timopheevii* is a valuable source of stem rust resistance. Several cultivars have been reported to confer strong resistance, such as LongReach Lancer, which confers *Sr36*-based stem rust resistance (Walkowiak et al., 2020), and an Australian prime hard wheat cultivar, Cook, which confers *Sr36* and *Yr18* (Bariana et al., 2001). The gene *Sr36* exhibits a near-immune reaction to races TTKSK and TTKST of *Puccinia graminis* f. sp. *tritici* at both the seedling and adult plant stages (Jin et al., 2009). In addition, resistance to powdery mildew, conferred by the *Pm6* and *Sr40* genes, was also mapped to chromosome 2G (Badaeva et al., 2000). In addition, a genome-wide association study showed that introgression lines carrying segments from *T. timopheevii* 2A were associated with significantly higher gluten content (Leonova et al., 2023). Our results showed that the line F9 carrying 2G segment was resistant to the leaf rust isolate used in this analysis, while another line (F5), also containing 2G segment, was resistant to the tested yellow rust isolate.

The combination of GBS and FISH has proven effective for characterizing genome structure and genetic diversity in the *T. timopheevii* group (Peng et al., 2023), supporting the usefulness of combined molecular and cytogenetic methods as applied in our study.

In conclusion, the line F9 showed reduced symptoms to the tested leaf rust isolate and had a higher number of spikelets per main spike than Mv9kr1 and the Mv Nádor elite wheat variety. The number of grains per main spike was also higher than the Mv9kr1 control. Line F5 showed reduced symptoms to the tested yellow rust isolate and had higher values for grain number per main spike and main spike weight than Mv9kr1. The line F2 was susceptible to both leaf and yellow rust isolates, but showed increased main spike length and significantly higher number of spikelets per main spike than Mv9kr1 or Mv Nádor. The number of grains per main spike was also significantly higher than in the case of Mv9kr1. At the same time, pre-breeding line A7 showed favorable yield-component traits but was susceptible to the tested rust isolates. Therefore, these lines represent promising pre-breeding materials for developing segregating populations, marker-trait validation, and further genetic dissection of rust resistance and favorable yield-component traits.

In general, our results demonstrate that GBS-based genome coverage analysis is an effective tool for characterizing complex introgression lines and for identifying homoeologous relationships and structural variation in wheat – *T. timopheevii* relative hybrids. The recurrent involvement of specific chromosomes, particularly 2B and 2G, provides valuable insight into genome compatibility and recombination behaviour, which is essential for the targeted exploitation of *T. timopheevii* genetic diversity in wheat breeding.

## Conclusion

A diverse wheat pre-breeding population potentially carrying *T. timopheevii* introgressions was analyzed using ddRAD-seq read-coverage profiling against the newly available *T. timopheevii* reference genome, followed by targeted FISH validation and phenotypic characterization. Multiple introgressed segments and structural rearrangements were detected, predominantly involving the B and G genomes. Field evaluation under the tested conditions indicated that some pre-breeding lines exhibited higher spikelet and grain numbers, suggesting the potential value of selected introgression lines for further pre-breeding validation. These results demonstrate the value of integrating genomic, cytogenetic, and phenotypic approaches for the characterization of wheat, *T. timopheevii* pre-breeding material.

## Data Availability

The datasets presented in this study can be found in online repositories: https://www.ebi.ac.uk/biostudies/arrayexpress/studies/E-MTAB-17094.
