# Supplementary material for: GBS-based identification of Triticum timopheevii introgressions in a wheat pre-breeding population
Source: Front Plant Sci. 2026 Aug 5;17:1887191. doi: 10.3389/fpls.2026.1887191 (PMC13486228; doi:10.3389/fpls.2026.1887191)
Supplement: Supplementary file 5 [file DataSheet5.pdf]

Journal name: Frontiers in Plant Science

Figure S5 for the manuscript entitled to 'GBS-based identification of *Triticum timopheevii* introgressions in a wheat pre-breeding population' written by Eszter Gaál*[1], Andrea Uhrin*†[1], Balázs Kalapos[1], Kateřina Holušová[2], Miklós Álmos Pance[1], Andrea Gulyás[1], Norbert Hidvégi[1], Jan Bartoš[2], Klaudia Kruppa[1], Edina Türkösi[1], Dimitar Douchkov[3], Moozhan Serpoush[3], László Ivanizs[1], András Farkas[1,4], Éva Szakács[1], István Molnár[1], Péter Mikó[1]

[1]Department of Biological Resources, Centre for Agricultural Research, Hungarian Research Network, Martonvásár 2462, Hungary

[2]Institute for Experimental Botany of the Czech Academy of Sciences, Centre of Plant Structural and Functional Genomics, Olomouc 77900, Czech Republic

[3]Leibniz Institute of Plant Genetics and Crop Plant Research (IPK), OT Gatersleben, Germany

[4]Research and Development Institute for Wildlife and Mountain Resources, Progresului nr 35/B, 530240, Miercurea Ciuc, Romania

*These authors contributed equally to this work

†corresponding author: uhrin.andrea@atk.hun-ren.hu

The file contains the normalized GBS read coverage of the pre-breeding lines F9, F8, B9, B7 and B8 along the A, B, D, A^t and G chromosomes of the *in silico* hybrid. Each panel corresponds to a different wheat chromosome (indicated in the upper part of each panel, Ta: *Triticum aestivum,* Tt: *Triticum timopheevii*). The x-axis represents the genomic position within the chromosome in Mb, while the y-axis shows the normalized read coverage in the case of the indicated chromosome.

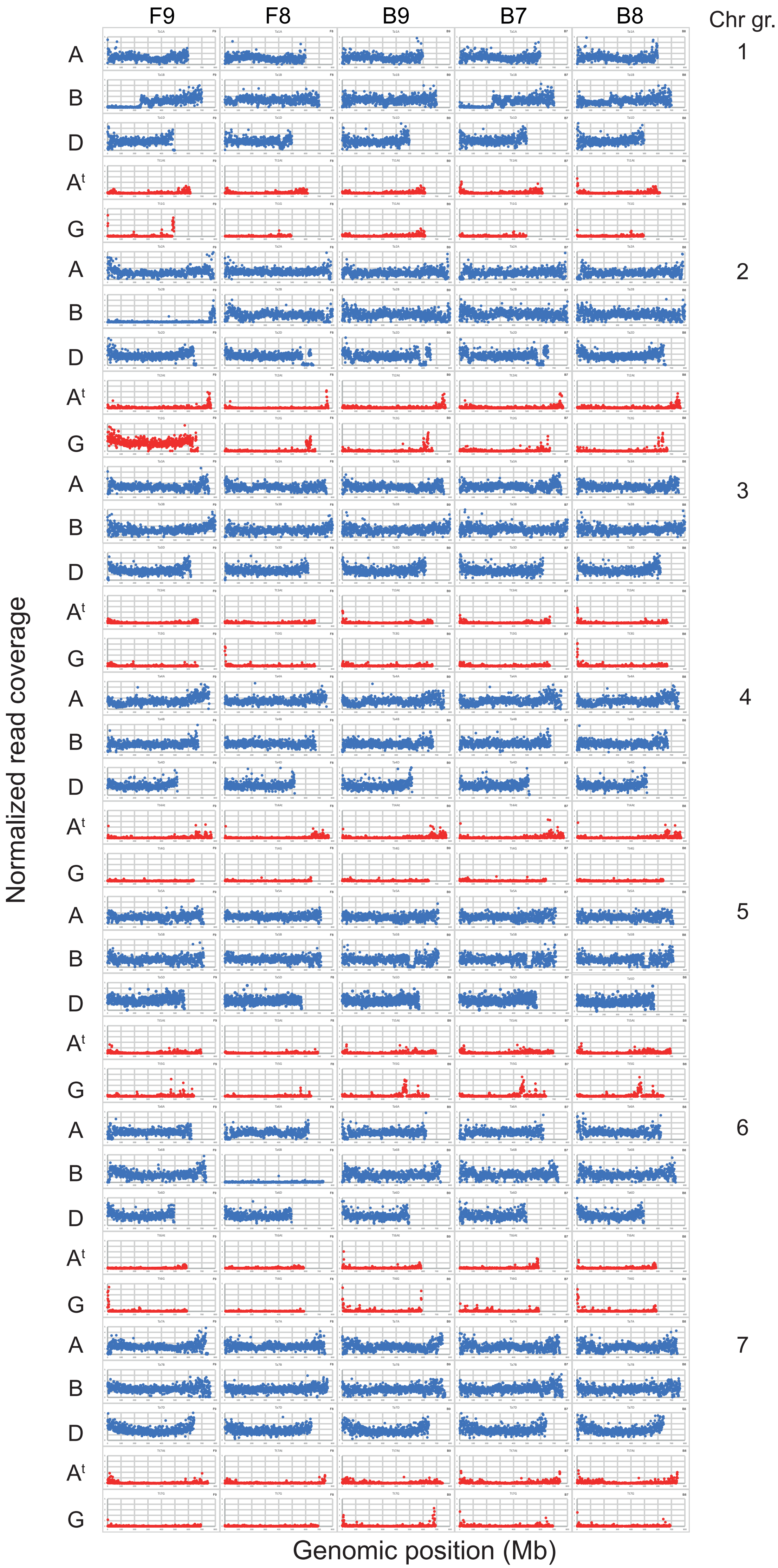

F9
F8
B9
B7
B8
Chr gr.
A
B
D
A^t
G
1
2
3
4
5
6
7
Normalized read coverage
Genomic position (Mb)
