# Supplementary material for: GBS-based identification of Triticum timopheevii introgressions in a wheat pre-breeding population
Source: Front Plant Sci. 2026 Aug 5;17:1887191. doi: 10.3389/fpls.2026.1887191 (PMC13486228; doi:10.3389/fpls.2026.1887191)
Supplement: Supplementary file 7 [file Table3.docx]

Journal name: Frontiers in Plant Science

Supplementary Table 3 for the manuscript entitled to 'GBS-based identification of Triticum timopheevii introgressions in a wheat pre-breeding population' written by:

Eszter Gaál^#1^, Andrea Uhrin^#*1^, Balázs Kalapos^1^, Kateřina Holušová^2^, Miklós Álmos Pance^1^, Andrea Gulyás^1^, Norbert Hidvégi^1^, Jan Bartoš^2^, Klaudia Kruppa^1^, Edina Türkösi^1^, Dimitar Douchkov3, Moozhan Serpoush^3^,László Ivanizs^1^, András Farkas^1,4^, Éva Szakács^1^, István Molnár^1^, Péter Mikó^1^

^1^ Hungarian Research Network (HUN-REN), Centre for Agricultural Research, Agricultural Institute, 2462 Martonvásár, Hungary

^2^ Institute for Experimental Botany, Centre of Plant Structural and Functional Genomics, Olomouc 779 00, Czech Republic

^3^Leibniz Institute of Plant Genetics and Crop Plant Research (IPK), OT Gatersleben, Germany

^4^Research and Development Institute for Wildlife and Mountain Resources, Progresului nr 35/B, 530240, Miercurea Ciuc, Romania

^#^ These authors contributed equally to this work

*Corresponding author: uhrin.andrea@atk.hun-ren.hu Orcid-ID: 0009-0002-7014-2138, phone: +3622569550

Supplementary Table 3. Morphological traits of the selected lines grown in field conditions.

|  | Length of main spike (cm) | Weight of main spike (g) | Grain weight of main spike (g) | No. of spikelets per main spike | No. of grains per main spike | Thousandgrain weight (g) |
| --- | --- | --- | --- | --- | --- | --- |
| Mv Nádor | 10,7 ± 1,1 | 4,1 ± 0,8 | 3,2 ± 0,7 | 19,1 ± 0,7 | 82,5 ± 11,1 | 38,6 ± 7,9 |
| Mv9kr1 | 9,8 ± 0,5 | 2,7 ± 0,5 | 2,3 ± 0,4 | 19,9 ± 1,3 | 56,3 ± 6,3 | 41,5 ± 8,4 |
| A7 | 12,3 ± 0,6**†† | 5,6 ± 0,6** | 4,9 ± 1,1**†† | 20,6 ± 1†† | 85,8 ± 6,7** | **57,2 ± 13,7*††** |
| D7 | 10,8 ± 0,9* | 3,6 ± 0,6** | 2,9 ± 0,5 | 20,7 ± 1,3† | 50,4 ± 8,5†† | **56,7 ± 2,4**††** |
| F1 | 11,2 ± 0,9** | 3,6 ± 0,2** | 2,5 ± 0,4 | 20,8 ± 1,6† | 47 ± 3,7**†† | **55,2 ± 7,9*††** |
| E7 | 10,2 ± 0,7 | 3,1 ± 0,4†† | 2,4 ± 0,3† | 19,6 ± 1 | 46,5 ± 3,6**†† | **51,7 ± 6,6*††** |
| E8 | 9,5 ± 0,7† | 3 ± 0,3†† | 2,3 ± 0,3† | 21 ± 1,3†† | 45,3 ± 4,6**†† | **51,7 ± 3,4**††** |
| B11 | 9,1 ± 0,6*†† | 3,8 ± 0,4** | 3,2 ± 0,3** | 19,8 ± 0,8 | 62,3 ± 8†† | **50,8 ± 3,4*††** |
| D6 | 9,9 ± 2,3 | 4,4 ± 0,7** | 3,5 ± 0,6** | 20,1 ± 0,7† | 71,8 ± 8,3** | **49 ± 6,4†** |
| A6 | 9,3 ± 0,8† | 5,2 ± 0,7** | 4,1 ± 0,6**† | 19,7 ± 1,1 | 84,5 ± 10** | **48,8 ± 5,4†** |
| B10 | 8,2 ± 0,9**†† | 2,6 ± 0,5†† | 2,1 ± 0,4†† | 17,3 ± 2,2* | 44,3 ± 7,8**†† | **48,2 ± 4,6†** |
| E11 | 10,6 ± 1 | 3,4 ± 0,5* | 2,7 ± 0,5 | 21 ± 1,3†† | 58,5 ± 6,4†† | **46 ± 4†** |
| C3 | 11,3 ± 1** | 3,7 ± 0,4 | 2,9 ± 0,4* | 20,1 ± 0,9† | 71,1 ± 6**† | 41,3 ± 5 |
| F8 | 9,5 ± 0,5† | 4 ± 0,8** | 3,2 ± 0,8* | 18,9 ± 1,2 | 76,8 ± 8,2** | 41,2 ± 7,1 |
| F5* | 10,6 ± 0,9 | 3,7 ± 0,4** | 2,9 ± 0,4* | 19,4 ± 1 | 75,5 ± 8,3** | 38,3 ± 4,4 |
| F9* | 9,9 ± 1 | 2,8 ± 0,8†† | 2 ± 0,7† | 24,5 ± 1,6**† | 62,5 ± 9,5†† | 32 ± 9 |
| F10 | 12,2 ± 0,9**† | 2,5 ± 0,6†† | 1,7 ± 0,4*†† | 27,2 ± 1,4**†† | 62,5 ± 6,6†† | 27,4 ± 5,3**†† |
| F2* | 14,3 ± 1,4**†† | 2,9 ± 0,8 | 2 ± 0,7†† | 27 ± 1,4**†† | 73,3 ± 12,9** | 26,5 ± 6,8**†† |

Values are the means ± standard deviations of ten measurements. The translocational lines are indicated with asterisk.

*Significantly different from the value of parental wheat Mv9kr1 at p=0.01.

**Significantly different from the value of parental wheat Mv9kr1 at p=0.001.

†Significantly different from the value of elite wheat variety MvNádor at p=0.01.

††Significantly different from the value of elite wheat variety MvNádor at p=0.001.
